# Supplementary material for: Assessment of the association between periodontal disease and total cancer incidence and mortality: a meta-analysis
Source: PeerJ. 2022 Nov 7;10:e14320. doi: 10.7717/peerj.14320 (PMC9648345; doi:10.7717/peerj.14320)
Supplement: Supplemental Information 4 [file peerj-10-14320-s004.docx]

1、The rationale for conducting the systematic review / meta-analysis

Periodontal disease is one of the most common oral diseases and is closely related to systemic diseases. In recent years, a number of epidemiological and clinical studies have revealed the correlation between periodontitis and cancers. Based on the cancer data published in the United States in 2019, the highest incidence and mortality of cancers included lung cancer, breast cancer, prostate cancer and rectal cancer, so we analyzed the correlation between periodontal disease and the incidence and mortality of four cancers.

2、The contribution that it makes to knowledge in light of previously published related reports, including other meta-analyses and systematic reviews

Firstly, we analyzed the correlation between periodontitis and four cancers from the occurrence and death of cancer, which was not found in other relevant reviews or meta-analyses. Secondly, we included more literatures and conducted subgroup analysis from multiple perspectives, which resulted in more reliable results and conclusions.

We believe that our study makes a significant contribution to the literature and that this paper will be of interest to the readership of your journal because to the best of our knowledge, our study is the first to perform such a comprehensive and detailed analysis, as we included all the latest studies and findings. Our results highlight the importance of increasing awareness regarding periodontal health and promoting measures to maintain good oral health. Moreover, the need for further studies on this subject has also been highlighted.
