# Supplementary material for: Assessment of the association between periodontal disease and total cancer incidence and mortality: a meta-analysis
Source: PeerJ. 2022 Nov 7;10:e14320. doi: 10.7717/peerj.14320 (PMC9648345; doi:10.7717/peerj.14320)
Supplement: Supplementary file 5 [file peerj-10-14320-s005.odt]

Table S1. Newcastle-Ottawa Scale for assessing the quality of studies in the meta-analysis

| Author（Year） | Selection |  | Comparability |  | Exposure | Qualiy score |
| --- | --- | --- | --- | --- | --- | --- |
| A | B | C | D |  | E |  | F | G | H |  |
| Hujoel et al 2003 | * | * | * |  |  | * * |  | * | * | * | 8 |
| Michaud et al 2008 | * | * | * |  |  | * * |  | * | * |  | 7 |
| Arora et al 2010 | * |  |  |  |  | * * |  | * | * | * | 6 |
| Ahn et al 2012 | * | * | * |  |  | * * | * | * | * | * | 9 |
| Mai et al 2014 | * | * |  |  |  | * * | * | * | * | * | 8 |
| Wen et al 2014 | * | * | * |  |  | * * | * | * | * |  | 8 |
| Chrysanthakopoulos et al 2016 | * | * | * |  |  | * * | * | * |  |  | 7 |
| Chung et al 2016 | * | * | * |  |  | * * | * | * | * |  | 8 |
| Freudenheim et al 2016 | * |  |  |  |  | * * | * | * | * | * | 7 |
| Mai et al 2016 | * | * | * |  |  | * * | * | * | * |  | 8 |
| Michaud et al 2016 | * | * | * |  |  | * * | * | * | * |  | 8 |
| Dizdar et al 2017 | * | * | * |  |  | * * | * | * | * |  | 8 |
| Han et al 2017 | * | * | * |  |  | * * | * |  |  |  | 6 |
| Lee et al 2017 | * | * | * |  |  | * * | * | * | * | * | 9 |
| Momen-Heravi et al 2017 | * | * | * |  |  | * * | * | * | * | * | 9 |
| Nwizu1 et al 2017 | * |  |  |  |  | * * | * | * | * | * | 7 |
| Sfreddo et al 2017 | * | * | * | * |  | * * |  | * | * | * | 9 |
| Chou et al 2018 | * | * |  |  |  | * * | * | * | * | * | 8 |
| Heikkilä et al 2018 | * | * | * |  |  | * * | * | * | * |  | 8 |
| Hu et al 2018 | * | * | * |  |  | * * | * | * |  |  | 7 |
| Lee et al 2018 | * | * | * |  |  | * * | * | * | * |  | 7 |
| Michaud et al 2018 | * | * | * |  |  | * * | * | * | * |  | 8 |
| Güven et al 2019 | * | * | * |  |  | * | * | * | * |  | 7 |
| Lu et al 2019 | * | * | * |  |  | * * | * | * |  |  | 7 |
| Tai et al 2019 | * | * |  |  |  | * | * | * | * |  | 6 |
| Yoon et al 2019 | * | * |  |  |  | * * | * | * | * |  | 7 |
| Huang et al 2020 | * | * | * |  |  | * * | * | * | * |  | 8 |

A: Is the case definition adequate; B: Representativeness of the cases; C: Selection of Controls; D: Definition of Controls; E: Comparability of cases and controls on the basis of the design or analysis; F: Ascertainment of exposure; G: Same method of ascertainment for cases and controls; H: Non-Response Rate.
